# Gap-Prepulse Inhibition of the Acoustic Startle Reflex (GPIAS) for Tinnitus Assessment: Current Status and Future Directions

**DOI:** 10.3389/fneur.2015.00088

**Published:** 2015-04-28

**Authors:** Alexander Galazyuk, Sylvie Hébert

**Affiliations:** ^1^Department of Anatomy and Neurobiology, Northeast Ohio Medical University, Rootstown, OH, USA; ^2^International Laboratory for Research on Brain, Music, and Sound (BRAMS), Faculty of Medicine, School of Speech Pathology and Audiology, Université de Montréal, Montréal, QC, Canada

**Keywords:** gap-prepulse inhibition of the startle reflex, tinnitus assessment, animal model of tinnitus, human tinnitus

## Abstract

The progress in the field of tinnitus largely depends on the development of a reliable tinnitus animal model. Recently, a new method based on the acoustic startle reflex modification was introduced for tinnitus screening in laboratory animals. This method was enthusiastically adopted and now widely used by many scientists in the field due to its seeming simplicity and a number of advantages over the other methods of tinnitus assessment. Furthermore, this method opened an opportunity for tinnitus assessment in humans as well. Unfortunately, multiple modifications of data collection and interpretation implemented in different labs make comparisons across studies very difficult. In addition, recent animal and human studies have challenged the original “filling-in” interpretation of the paradigm. Here, we review the current literature to emphasize on the commonalities and differences in data collection and interpretation across laboratories that are using this method for tinnitus assessment. We also propose future research directions that could be taken in order to establish whether or not this method is warranted as an indicator of the presence of tinnitus.

The perceptual phenomenon of tinnitus, commonly described as ringing in the ears, is an affliction that affects nearly one-third of Americans (1, 2). Although significant progress has been made in the last few decades, the neural basis of tinnitus is still poorly understood. Several hypotheses on the brain mechanisms responsible for tinnitus development have been raised. Testing of these hypotheses requires a reliable objective measure of tinnitus in animal models and humans. A number of animal models have been developed over the years [for a review, see Ref. (3)]. These models have used various behavioral training techniques including lick or lever pressing suppression (4–8), two-choice operant conditioning (9–11), and reflex modification (12). The latter paradigm exploits the acoustic startle reflex present in all mammals, which consists of contraction of the major muscles of the body following a loud and unexpected sound (13). The reflex is reduced when preceded by a silent gap embedded in a soft background noise or tone. The ratio between the magnitude of the startle stimulus presented alone (no-gap trial) and trials in which a gap preceded startle stimulus (gap trials) is calculated as the gap-prepulse inhibition of the acoustical startle (GPIAS) ratio (12) and reflects gap detection. Reduced inhibition by gaps embedded in specific background noise frequencies is assumed to reflect tinnitus frequency: because tinnitus is qualitatively similar to the background noise, it “fills-in” the gap and hence, reduces inhibition.

Because of its simplicity, a wide range of researchers has enthusiastically adopted the paradigm to study tinnitus. However, recent studies in tinnitus animal models and humans have seriously challenged this “filling-in” assumption to the point of questioning whether the method is worthy of further pursuit. As a result, the field of tinnitus research split into two coexisting camps of scientists: one camp continues using GPIAS method for tinnitus assessment because researchers still believe that this method is a reliable technique for tinnitus assessment whereas another camp decided to stay away from this method because researchers do not think GPIAS is detecting tinnitus in animals or humans.

The main purpose of this review is twofold. First, by analyzing the large GPIAS literature we will extract the most common standards for GPIAS data collection and analysis that would permit standardized parameters. It will allow data comparison across laboratories and might also help to collect definitive arguments to support the idea that GPIAS indeed assess tinnitus. Second, analyzing literature from the opposing camp we will try to outline the research directions that might help to do the final verification of the validity of GPIAS for tinnitus assessment.

## Advantages and Problems with the GPIAS Method

The GPIAS model has several advantages over the other tinnitus paradigms. Since it is based on a reflex, the method is much cheaper and simpler than other methods requiring training animals for weeks or months [e.g., Ref. (14, 15)]. It also allows for tinnitus screening of a large number of animals: testing of animals before and after tinnitus induction can help separate tinnitus positive from tinnitus negative animals. The seeming simplicity of and a possibility to be used on human subjects made GPIAS the most widely used method for tinnitus assessment especially among scientists with little experience in animal behavior. Yet, the seminal publication of Turner and colleagues mainly introduced a proof-of-principle to use GPIAS for tinnitus assessment rather than setting rigorous standards for the method. Furthermore, the commercially available equipment designed for GPIAS assessment generally provides a hardware/software kit leaving on users to setup their own standards and criteria to decide what changes in the gap detection performance can be interpreted as an indication of tinnitus and what are not. Therefore, different scientists used their own criteria to interpret gap detection data and often these criteria can vary dramatically from one lab to the other. Table 1 summarizes the main characteristics of studies that have used the GPIAS paradigm across species, namely, rats (12, 16–34), mice (35–41), guinea pigs (42–45), gerbils (46), hamsters (47), and humans (48–52).

**Table 1:** **Main methodological characteristics of animal studies that have used GPIAS and human studies that have examined gap detection for tinnitus assessment**.

	Authors/group	Species/number	Method for tinnitus	Stimuli	Gap duration	Time tested	Criterion for deficit
**Rats**			**Noise exposure**				

	Turner et al. (12)	31 Long–Evans 16 NE + 16 controls	NBN 16 kHz for 1 h at 116 dB SPL meant to induce a 10 kHz tinnitus	60 dB SPL Background: BBN, NBN at 10 or 16 kHz SS 20 ms at 115 dB SPL 10 Startle-only + 12 Gap trials	50 ms ending 50 ms before startle	Pre and every week post NE for 2 months	Signif. GPIAS reduction vs. control

	Wang et al. (16)	29 Fisher-Brown Norway 14 NE + 15 controls	NBN 17 kHz for 1 h at 116 dB SPL	60 dB SPL Background: BBN, NBN at 4, 8, 10, 12, 16, 20, 24, 32 kHz SS 20 ms at 115 dB SPL/number of stim NS	50 ms ending 50 ms before startle	20 days post NE every 2 weeks up to 16 weeks	Signif. GPIAS reduction vs. control

	Kraus et al. (17)	12 Sprague-Dawley 9 NE + 3 controls	NBN 12 kHz for 2 h at 126 dB SPL	60 dB SPL background NBN at 6, 12, 16, 20, and 24 kHz 20 pairs of Gap and SS trials	50 ms ending 50 ms before startle	Pre NE + days 1–10 post weeks 8–10 post	A lack of GPIAS

	Luo et al. (19)	6 Long–Evans	10 kHz tone for 3 h at 116 dB SPL	60 dB SPL background BBN + NBN 6–8; 10–12; 14–16; 18–20; 26–28 kHz SS 50 ms at 115 dB SPL/8 SS trials + 8 gap trials	40 ms	Not specified	Signif. GPIAS reduction vs. control

	Pace and Zhang (18)	29 Long–Evans	10 kHz tone for 2 h at 118–120 dB SPL second exposure 5 weeks later for 3 h	60 dB SPL background BBN + NBN 6–8, 10–12, 14–16, 26–28 kHz SS 50 ms at 115 dB SPL/8 SS + 8 gap trials	40 ms ending 50 ms before startle	1 day post NE and biweekly for 6 weeks	Signif. GPIAS reduction vs. control

	Engineer et al. (20)	28 Sprague-Dawley	NBN 16 kHz for 1 h at 115 dB SPL	65 dB SPL NBN at 2, 4, 8, 10, 16, 20, 24 kHz SS 20 ms white noise burst at 100 dB SPL 15 SS and 15 Gap trials	50 ms ending 50 ms before startle	4 weeks 10 and 20 days after beginning of the therapy	Signif. GPIAS reduction vs. control

	Holt et al. (21)	24 Sprague-Dawley 8 NE, 8 SA, 8 controls	NBN 10 kHz for 4 h at 118 dB SPL (*n* = 6) SA: 300 mg/kg ip. (*N* = 6 animals)	Background tones 4, 8, 12, 16, 20, and 24 kHz 45 and 60 dB SPL and 75 and 90 for NE rats SS 20 ms at 120 dB SPL/10 trials each frequency	50 ms ending 50 ms before startle	S: 2 h previous to imaging N: 24 h after NE 2 h prior to imaging	Signif. GPIAS reduction vs. control

	Ropp et al. (22)	Sprague-Dawley	16 kHz tone for 2 h at 116 dB SPL	50 dB SPL NBN, 7, 10, 14, 20 kHz SS BBN 20 ms at 110 dB SPL/12 trials	30 ms ending 50 ms	Two to three times a week, 1–4 months post NE	Signif. GPIAS reduction vs. control

	Norman et al. (23)	10 Sprague-Dawley	17 kHz tone for 2 min at 115 dB SPL	70–85 dB SPL Background: NBN at 10 or 16 kHz SS 20 ms at 90–100 dB SPL 20 Startle-only + 20 Gap trials	50 ms ending 50 ms before startle	10–20 min after exposure	Signif. Increase in tinnitus index vs. control

			**Salicylate**				

	Turner and Parrish (24)	10 Fisher-Brown Norway	Salicylate 0–150–250–300–350 mg/kg	60 dB SPL BBN or NBN at 4, 8, 10, 12, 16, 20, 24, 32 kHz PPI in quiet 50 ms pulse at 60 dB SPL at same frequencies	50 ms ending 50 ms before startle	Pre and 2 h post and 1 week post (washout)	Signif. GPIAS reduction vs. control

	Sun et al. (25)	16 Sprague-Dawley 10 SA, 6 SA + vigabatrin	Salicylate 250 mg/kg	70 dB SPL BBN 3 gap types: onset, offset, onset-offset SS 20 ms at 100 dB SPL/20 trials in each gap condition	50 ms ending 40 ms before startle	pre and post 1 h, 3 h, and 1 day post	N/S

	Yang et al. (26)	20 Sprague-Dawley 10 startle, 10 SIPAC	Salicylate 150 or 250 mg/kg	SIPAC: 16 kHz tone or 2, 4, 8, 16, 20, NBN at 40–60 dB SPL GPIAS: 60 dB SPL NBN at 6, 12, 16 kHz SS BBN at 115 dB SPL/20 pairs at each frequency (X3)	50 ms ending 50 ms before startle	1 h after injection Startle 2 h after injection GPIAS	Signif. GPIAS reduction vs. control
	Ralli et al. (27)	24 Sprague-Dawley 12 SA, 12 quinine	Salicylate 300 mg/kg/day 4 days quinine 200 mg/kg/day 4 days	60 dB SPL NBN at 6, 12, 16, 20 kHz SS BBN at 50 ms 117 dB SPL/100 gap and 100 SS trials	100 ms ending 50 ms before startle	2 h after each treatment (4 days) 24 h the last treatment	Signif. GPIAS reduction vs. control

	Su et al. (28)	6 Wistar	Salicylate 350 mg/kg	60 dB SPL NBN 6, 12, 16, 20, 24 kHz SS NBN 5–10 kHz at 105 dB SPL 100 pairs of gap and SS trials; 20 of each Freq	75 ms ending 25 ms before startle	1 h after injection	Signif. GPIAS reduction vs. control

	Ralli et al. (29)	36 Sprague-Dawley 12 SA, 12 MEM, 12 SAL + MEM	Salicylate 300 mg/kg 5 mg/kg/day	60 dB SPL NBN at 6, 12, 16, 20 kHz SS BBN 50 ms at 117 dB SPL 80 gap and 80 no-gap trials	100 ms ending 100 ms before startle	2 h after each treatment (5 days)	Signif. GPIAS reduction vs. control

	Hu et al. (30)	48 Sprague-Dawley	Chronic salicylate: 200 mg/kg/day for 3, 7, or 14 days Acute salicylate 400 mg/kg	65 dB SPL NBN 6, 12, 16 kHz SS WN at 100 dB SPL 30 gap and 30 SS trials	50 ms ending 50 ms before startle	1 h before sacrifice	Signif. GPIAS reduction vs. control

	Park et al. (31)	14 Sprague-Dawley	Salicylate 400 mg/kg for 8 days	60 dB SPL NBN at 16 kHz SS BBN 20 ms at 120 dB SPL 20 gap and 20 SS trials	50 ms ending 50 ms before startle	Pre and 2 h post NE every 24 h during 9 days	Signif. GPIAS reduction vs. control

			**Blast exposure**				

	Mahmood et al. (32)	24 Sprague-Dawley 10 blast-exposed + sidenafil 6 blast-exposed + saline, 8 sidenafil	3 Blast exposure shock waves	60 dB SPL NBN 6–8; 10–12; 14–16; 18–20; 26–28; BBN; SS BBN 50ms at 115 dB SPL PPI 40 ms at 60 dB SPL	40 ms ending 50 ms before startle	1 h post blast	Signif. GPIAS reduction vs. control

	Luo et al (33)	39 Sprague-Dawley	Single 10 ms blast 22 psi with one ear occluded with silicone earplug	60 dB SPL NBN 6–8; 10–12; 14–16; 18–20; 26–28; BBN; SS BBN 50ms at 115 dB SPL	40 ms	1 day, 1 month, 3 months	Signif. GPIAS reduction vs. control

	Luo et al. (34)	39 Sprague-Dawley	Single 10 ms blast 22psi with one ear occluded with silicone earplug	60 dB SPL NBN 6–8; 10–12; 14–16; 18–20; 26–28; BBN; SS BBN 50 ms at 115 dB SPL	40 ms	1 day, 1 month, 3 months	Signif. GPIAS reduction vs. control

**Mice**			**Noise exposure**				

	Llano et al. (35)	13 CBA/Caj aged 6 NE, 4 sham, 3 controls	NBN 16 kHz for 1 h at 116 dB SPL	60 dB SPL NBN at 4, 8, 10, 12.5, 16, 20, 24, 32 Hz + BBN PPI NBN 50 ms at 60 dB SPL number of stimuli NS	50 ms ending 50 ms before startle	Pre and post NE and 24–30 months	Group differences

	Turner et al. (36)	C57B16 mixed 15 NE, 8 sham	NBN 16 kHz for 1 h at 116 dB SPL	60 dB SPL NBN at 4, 8, 10, 12, 16, 20, 24, 32, BBN gap and SS: 290 trials	50 ms ending 50 ms before startle	Pre; day 1, 3–4, 7–8 weekly for 12 weeks post NE then monthly for 7 months	Signif. GPIAS reduction vs. control

	Longenecker and Galazyuk (38)	22 CBA/CBJ 14 NE, 8 controls	NBN 16 kHz for 1 h at 116 SPL	75 dB SPL NBN 10, 12.5, 16, 20, 25, 31.5 kHz SS WN 20 ms at 110 dB SPL 10 gap and 10 SS trials at each frequency	20 ms ending 80 ms before startle	Pre NE; day 1, 3–5, 7 days post NE weekly for 2 months; at 3 months	Signif. GPIAS reduction vs. control
	Longenecker et al. (37)	CBA/CaJ follow-up to above study	NBN 16 kHz for 1 h at 116 SPL	Idem	20 ms ending 80 ms before startle	6–12 months post NE	Signif. GPIAS reduction vs. control

	Hickox and Liberman (39)	CBA/CaJ	NBN 8–16 kHz for 2 h at 100 or 94 dB SPL	Near gap and far gap 60 dB SPL BBN and NBN from 5.6 to 45.3 kHz SS 20 ms BBN continuous 60 dB SPL/11 blocks of trials	50 ms ending 0 or 80 ms before startle	1–10 weeks post NE	Signif. GPIAS reduction vs. control

	Middleton et al. (41)	CBA/CaJ	NBN 16 kHz for 45 min at 116 dB SPL	70 dB SPL NBN at 10, 12, 16, 20, 24, and 32 kHz SS 20 ms BBN at 115 dB SPL	50 ms ending 80 ms before startle	Pre; 2–9 weeks post NE	Fixed GPIAS threshold

	Li et al. (40)	IRC (CD-1)	NBN 16 kHz for 45 min at 116 dB SPL	70 dB SPL NBN 10, 12, 16, 20, 24, 32 kHz SS, NS	50 ms ending 80 ms before startle	Pre; 1 week post NE	Signif. GPIAS reduction vs. control

**Guinea pigs**							

	Dehmel et al. (42)	14 pigmented 7 NE + 7 sham	NBN 7 kHz for 2 h at 97 dB SPL 2 exposures	60 and 70 dB SPL NBN 4–6, 8–10, 12–14, 16–18 kHz + BBN SS 20 ms BBN at 115 dB SPL/10 pairs of for each frequency	15 ms ending 85 ms before startle	Pre; 2–3 weeks post NE	Signif. GPIAS reduction vs. control

	Koehler and Shore (43)	16 pigmented 10 NE + 6 sham	NBN 7 kHz for 2 h at 97 dB SPL 2 exposures	60 and 70 dB SPL NBN 4–6, 8–10, 12–14, 16–18 kHz + BBN, SS 20 ms BBN at 115 dB SPL	50 ms ending 50 ms before startle	Pre; 2 h post NE + biweekly	Signif. GPIAS reduction vs. control

	Mulders et al (44)	32 pigmented 20 NE + furosemide	NBN 10 kHz for 2 h at 124 dB SPL	60 or 70 dB SPL NBN 8 or 14 kHz 8 is below HL; 14 is within SS 20 ms NBN at 115 dB SPL/25 pairs of Gap and SS trials	50 ms ending 50 ms before startle	Pre; weekly testing post NE	Signif. GPIAS reduction vs. control

			**Salicylate**				

	Berger et al. (45)	24 pigmented	Salicylate 350 mg/kg	55, 60, or 70 dB SPL NBN 5, 9, 13, 17 kHz + BBN SS 20 ms BBN adjusted for each GP 95, 100, or 105 dB/10 pairs of gap and SS trials	50 ms ending 50 ms before startle	Pre; 2, 5, and 72 h post NE	Signif. GPIAS reduction vs. control

**Gerbils**			**Noise exposure**				

	Nowotny et al. (46)	9 Mongolian	NBN 10 kHz for 1 h at 105 dB SPL	65 and 75 dB SPL NBN 4–20 kHz SS BBN 20 ms optimal level 65–115 dB SPL, 25 pairs of gap and no-gap	50 ms ending 100 ms before startle	Pre + 3–5 weeks post NE	Signif. GPIAS reduction vs. control

**Hamsters**							

	Chen et al. (47)	18 hamsters 9 NE + 9 unexposed	10 kHz tone for 4 h at 115 dB SPL ± 6 dB	70–75–80 dB SPL NBN 12–14 kHz SS 20 ms BBN 100–105–110–115 dB SPL 15 trials	50 ms ending 100 ms before startle	2 weeks post NE and on until 28 weeks	N/S
**Humans**							

	Fournier and Hébert (48)	15 tinnitus + 15 control adults Clinical normal hearing 250–8 kHz L at 12.5, 14, and 16 kHz	Psychoacoustic assessment high-frequency tinnitus (11 and 16 kHz) Loudness 0.8 dB SL and 10.1 (11.3 and 16)	65 dB SPL NBN centered around 500 Hz and 4 kHz, SS BBN 50 ms at 105 dB (A) SPL	50 ms ending 120 ms before startle	Mean TD = 9.3 years	Group differences

	Campolo et al. (49)	13 tinnitus + 13 control adults important hearing loss and variability in tinnitus adults	Selection of NBN closest to tinnitus pitch tinnitus pitch at the center F of selected NBN tinnitus loudness match	15 dB SL NBN 1 octave above and below NBN tinnitus between 1 and 16 kHz Continuous background with gaps at random/GO/NO GO task	50 ms	Unknown	Group differences

	Mahmoudian et al. (50)	28 tinnitus + 33 control adults H < 20 dB HL 250–2 kHz < 40 dB HL 4–8 kHz	Psychoacoustic assessment of pitch, loudness, MML, and RI not used in the study	65 dB SPL, 75 ms pure tone at 500, 1, and 1.5 kHz with a gap within the tone	7 ms	Mean TD = 5 years	Group differences

	Mehdizade Gilani et al. (51)	20 tinnitus + 20 control adults all with HL ≤20 dB HL from 250 to 8 kHz Group differences not specified	Psychoacoustic assessment of pitch and loudness and MML not used in the study	Gap-in-noise (GIN) test 50 dB SL background noise Identification of 4/6 gaps of shortest gap is the threshold	2–6, 8, 10 12, 15, 20 ms	Median TD = 7 years	Group differences

	Boyen et al. (52)	22 tinnitus + 20 control adults No H differences in HL between groups	Psychoacoustic tinnitus pitch matching Loudness matching on a VAS 0–10	5, 10, and 25 dB SL 300 ms NBN of 4–8, 4–5, and 5–6.3 kHz 3I-3AFC with 1 interval containing a gap Adaptive procedure 2 down–1 up	Starting at 30 ms	Mean TD = 7 years	Group differences

*All animal studies have used unilateral noise-exposure except Engineer et al. (20) and Chen et al. (47) and while animals were under anesthesia*.

*BBN, broadband noise; NBN, narrow-band noise; SS, startle stimuli; NE, noise exposure; SA, salicylate; NS, not specified; TD, tinnitus duration; F, frequency; MEM, memantine*.

## GPIAS in Animal Models

First introduced by Turner and colleagues in 2006 using the noise-induced rat tinnitus model, the effectiveness of the GPIAS method in detecting tinnitus was further supported 1 year later via the salicylate-induced tinnitus rat model (26). This method of tinnitus assessment is currently one of the most utilized in the field. Fortunately, the method was developed by scientists with many years of intense experience and expertise in behavioral sciences. On the other hand, many basic but important aspects of this method received little attention in these two publications. Many scientists, especially those who have little experience with behavioral experiments, mistakenly believe that this is an easy and precise method for tinnitus screening and furthermore that it can be applied to practically any laboratory animal. As a result, many aspects of this methodology related to data collection and interpretation have been interpreted freely and thus vary across laboratories. Such diversity has made intra-species comparisons dubious at best, while inter-species data are unable to be compared at all. In our review, we want to avoid judging which of these diverse criteria are right or wrong. Instead, we review GPIAS literature in order to find consistencies and inconsistencies across laboratories in data collection and interpretation. In some instances, however, we would like to emphasize on some critical points of the methodology raised by selected publications, which, we believe, may be important for further method improvement.

## Tinnitus Induction

Methodologies of tinnitus induction in laboratory animals are not directly related to GPIAS. However, issues concerning these methods should be discussed here for the following reasons. First, understanding the pros and cons of various tinnitus induction techniques can assist researchers in choosing the most appropriate method for a given tinnitus study. Second, standardized methods for tinnitus induction, especially for the same animal model, may ameliorate data comparison across laboratories. There are two methods of tinnitus induction used in the field, namely, pharmacological manipulations and exposure to loud sounds (see Table 1 for a summary). Pharmacological methods encompass the effects of two drugs, salicylate and quinine. Salicylate has been proven to induce tinnitus in the vast majority of treated animals. Most of salicylate studies were performed on rats. After 1–2 h following systemic salicylate injection ranging in dosage from 150 to 400 mg/kg rats exhibit GPIAS deficits typically around 16 kHz (26), rarely at wider range of frequencies (24) or across all frequency range tested (28). In one salicylate study conducted on guinea pigs, GPIAS deficits were detected at slightly lower frequency range compared to rats, that is, between 8 and 10 kHz (45). Apparently, the dose of salicylate is not so critical for the outcome, because tinnitus has been reliably induced by lower as well as high doses. One of the main advantages of salicylate-induced tinnitus is its potential reversibility. Typically, the GPIAS deficits in salicylate models reverse back to normal within 72 h of the last administration. Although this method can reliably induce tinnitus in laboratory animals it has some disadvantages. First of all, salicylate has significant effects not only on periphery but also direct effect on the entire central auditory system [see Ref. (53) for a review]. Most importantly, the salicylate model has minimal relevance to human pathology, which is usually triggered by noise trauma, and does not induce chronic tinnitus, as tinnitus retreats when the intake is stopped.

Acoustic trauma is the most popular method of tinnitus induction for different tinnitus animal models. Although this method is the most common across laboratories, the parameters for inducing tinnitus are quite variable. Sound levels used for acoustic trauma vary from 94 dB SPL to 125 dB SPL. However, most laboratories follow the tinnitus induction protocol first described by Turner and colleagues (12). Despite very little justification in the original publication, the 116 dB SPL sound intensity level is commonly used for rats and mice (22, 36, 38, 41). But the intensities also range from 94 (39) to 126 dB SPL (8). For other animal tinnitus models such as guinea pigs, gerbils, and hamsters, sound intensity is slightly weaker, with 97, 105, and 115 dB SPL, respectively (46, 47, 54). Despite the differences in sound intensity some common themes exist between studies. The most common parameters for tinnitus induction include narrow-band-noise (often 1 octave bandwidth) presented during 1–2 h. In some studies, however, 1/3 octave or pure tones have been used. One of the most consistent features is unilateral noise exposure. This is an understandable commonality because the GPIAS method requires at least one normally functioning ear, while acoustic trauma may alter animal’s hearing performance.

Military personal are exposed to intense blasts of noise on a regular basis, which commonly result in an instantaneous perception of tinnitus. More than half of military personnel reported tinnitus following blast exposure [for a review, see Ref. (55)]. Although both noise and blast exposures have been classified in a similar way, blast exposure should be differentiated from noise exposure based on the following criteria. First, peak sound intensities during a blast can momentarily approach 200 dB SPL, a value starkly differentiated from noise exposure, which rarely exceeds 120 dB SPL, albeit for a longer duration. Second, damage to the auditory periphery can be extensive in blast-induced trauma. Disarticulation of the ossicular chain, perforation of the tympanic membrane, and significant structural damage to the organ of Corti are typical for blast-induced-trauma. Such damage is uncommon following noise exposure. In addition, traumatic brain injury and damage to internal organs are also typical following a blast exposure. Both the morbidity and prevalence of blast-induced tinnitus in military personnel would suggest that a blast-induced tinnitus animal model is important for tinnitus research. However, the extensive consequential damage resulting from blast exposure would make the development of such a tinnitus animal model very challenging. For these reasons, this model is only being developed in one laboratory (32–34, 56). These studies have suggested that after a single or multiple unilateral blast exposures animals typically developed gap detection deficits, which are evident across a wide range of sound frequencies.

In summary, there are several methods available to induce tinnitus in laboratory animals. Each of these methods has pluses and minuses that need to be carefully considered before implementation in a given animal tinnitus model.

## Criteria for the Presence of Tinnitus

GPIAS studies lack common criteria for tinnitus identification. Agreement between scientists on these criteria is vital for the field because these are the only measure of tinnitus we have to identify animals that experience tinnitus following tinnitus induction and to conclude whether tinnitus is affected by our manipulations. One of the possible reasons for a lack of a common criterion is that Turner et al. in their original publication (2006) did not separate sound exposed animals between tinnitus positive and tinnitus negative using the GPIAS method. The presence of tinnitus in the sound exposed rats had first been assessed using Bauer and Brozoski’s methods [see Ref. (5, 14, 57)] and then confirmed by GPIAS. Therefore, it is likely that different criteria have been designed by scientists who adopted the GPIAS method in their laboratories. Below, we provide an overview of tinnitus assessment criteria in attempt to find similarities across laboratories and also understand which of these criteria can be potentially used as a gold standard(s) in the field.

Unfortunately, some papers did not provide their criteria by which animals were separated on tinnitus positive and tinnitus negative after tinnitus induction. From the papers that did, we get the following picture. A majority of laboratories use a statistically significant reduction in GPIAS as a criterion for behavioral evidence of tinnitus. Although sometimes not clearly stated in the methods of published work, this criterion is most commonly used for identifying animals with gap detection deficits and/or for the separation of tinnitus positive from tinnitus negative individuals (16, 18, 20, 26, 30, 33, 34, 36–40, 45, 56, 58–60). These reductions in GPIAS typically were observed at a narrow frequency range in salicylate (21, 26, 30) as well as in noise-induced tinnitus animal models (37, 38, 41, 43, 44). Other laboratories established different strategies to assess whether animals developed tinnitus. One of these approaches uses a lack of GPIAS as a criterion to test whether an animal developed tinnitus (17, 44). In other words, the startle amplitude in “no-gap” and “gap” trials are compared statistically. If they are not significantly different in some animals, such animals are assigned to the tinnitus positive group. Some labs choose to not separate animals on tinnitus positive or negative. The GPIAS values of exposed animals are compared with a control (unexposed) group assuming that some of the animals in the exposed group would develop tinnitus following tinnitus induction (35). One more approach uses a fixed threshold above which animals would be considered as a tinnitus positive (41). In these studies, only mice that showed the GPIAS ratios under 0.65 are used for the control and noise-induced groups. After sound exposure, only mice that showed GPIAS ratios above 0.7 are included in the tinnitus positive category. One laboratory decided to stay away from GIPAS ratio as most researchers do but, instead, developed their own metric of tinnitus, tinnitus index (23).

Thus, a variety of criteria have been established to assess GPIAS deficits in laboratory animals. Each of these criteria has its own advantages and disadvantages and cannot be easily rejected or accepted as a gold standard. However, it is important for the field to choose one to allow data comparison across labs. Logically, it would be reasonable to focus on the most commonly used method: the statistically significant reduction in GPIAS relative to the control recorded before tinnitus induction.

## GPIAS and Hearing Loss

Hearing loss can potentially affect GPIAS screening for two reasons. First, hearing loss caused by noise exposure or ototoxic drugs can make the background sound and the embedded gaps less audible. If so, the GPIAS method would be impossible to use for tinnitus screening. Second, hearing loss can also attenuate startle reflex magnitude. Indeed, many sound exposed animals show a significant reduction (about 50%) of their startle response amplitude compared to the controls (8, 20, 38, 47). Such a reduction in startle amplitude can be observed up to a year after sound exposure (37). The continuous background noise during GPIAS testing has also been shown to substantially reduce startle response amplitude in a frequency dependent manner even in control (unexposed) animals (58). The GPIAS paradigm requires a robust startle reflex to observe its inhibition. If a startle is “overly suppressed” by hearing loss, this can result in a “floor effect,” which may mask any further suppression by a gap in the GPIAS test. This problem can be corrected by a slightly enhancing the startle stimulus intensity in noise-exposed mice (58). Since GPIAS always uses “no-gap”/“gap” startle ratios the absolute startle magnitude would not affect the ratio within a reasonable range of startle stimulus intensities (58). An alternative approach has been recently proposed to address startle suppression after tinnitus induction. Acoustic startle stimulus was substituted with a rapid air puff applied to the animal’s back that cannot be subject to hearing loss (8). This approach can definitely address noise-induced startle reductions, but it would not make the background sound more audible.

Another common approach to overcome the possible effect of hearing loss on GPIAS testing is unilateral sound exposure. This approach was adopted by the vast majority of laboratories that are using GPIAS for tinnitus assessment. Unilateral exposure preserves one ear for the subsequent testing and also allows the animal to serve as its own control. However, we have to keep in mind that the ascending auditory pathways are binaural. Therefore, it is reasonable to assume that a unilateral acoustic trauma might affect both contra- and ipsilateral sides. This hypothesis has been partially supported by the fact that the startle reflex magnitude is reduced in unilaterally exposed animals.

Pre-pulse inhibition (PPI) of the acoustic startle reflex is a widely accepted method to assess whether tinnitus induction manipulations have resulted in hearing loss in laboratory animals. During PPI experiments, a short duration noise-burst of the same amplitude as the background sound used in GPIAS testing is presented prior to the startle stimulus. It is assumed that if the pre-pulse reliably inhibits the startle reflex, the audibility of the background and the embedded gap is not an issue. Multiple lines of evidence suggest that this assumption is not accurate. First, if PPI could simply be thought of as the inverse of the GPIAS methodology, the amount of startle reflex suppression between these two approaches would be similar. Contrary to this notion, practically all GPIAS studies have demonstrated that the PPI is much more robust than GPIAS in sound exposed as well as in control (unexposed) animals [as well as in humans, e.g., Ref. (48)]. Second, the brain circuitries responsible for PPI and GPIAS are quite different. Several studies have shown that the auditory cortex is necessary for detections of gaps that are used for tinnitus assessment with GPIAS [e.g., Ref. (61–63)]. On the other hand, PPI does not require cortex (62, 64–66), indicating that PPI primarily reflects an automatic process at the pre-attentive stage. These issues raise concern to whether it is appropriate to use PPI to conclude that the animal is able to appropriately perceive background narrow-band noise presented in GPIAS testing.

## Testing of Tinnitus “Filling-In” Assumption in Tinnitus Animal Models

Two recent animal studies have investigated the tinnitus “fill in” hypothesis in rodent tinnitus models (39, 67). One study demonstrated that GPIAS deficits in noise-exposed mice were largely dependent on the interval between the silent gap and startle stimulus (39). These animals demonstrated GPIAS deficits only when the gap was placed immediately before the startle stimulus. This result is contradicting the “filling-in” assumption, because the presence of such deficits should be independent on where the gap is placed. The reversed gap detection performance between control and sound exposed animals over the entire frequency range for near-gaps vs. far-gaps strongly suggest that these different gap locations might suppress startle responses via fundamentally different mechanisms [see Figure 8 in Ref. (39)]. This may explain why animals with tinnitus would demonstrate different GPIAS deficits for near- and far-gaps. Rather the type of neurons, for instance, the gap termination neurons, might also play an important role in these findings (61). Another study tested the assumption using go/no-go operant gap detection task (67). This study found that the behavioral threshold for gap detection embedded into continuous narrow-band background noise is not different in rats before and after tinnitus induction with overdose of salicylate. Taken together results of these two studies suggest that data interpretation of GPIAS experiments should be taken with caution and raise the possibility that the mechanisms involved are less straightforward than was originally hypothesized.

## Gap Detection in Humans with Tinnitus

Five human studies have examined whether gap detection is impaired in tinnitus. These studies are discordant among themselves and also from animal studies in several ways (see Table 1). Two studies are compatible with gap detection impairment in tinnitus. Fournier and Hébert (48) used the GPIAS procedure as in Turner and Parrish (24) and was very similar. Gap trials consisted of 50 ms gaps embedded in high-frequency (centered around 4 kHz) and low-frequency (centered around 500 Hz) background noises set at 65 dB SPL. Startle noises were 50 ms broadband noise bursts (20 Hz–20 kHz) set at 105 dB (A) SPL. Prepulse inhibition trials consisted of 50 ms bursts of high- or low-frequency noises at 65 dB (A) SPL in a silent background followed by a startle sound. Participants had bilateral tinnitus for about 10 years and had normal clinical hearing from 250 Hz to 8 kHz not different from controls’ (thresholds only differed from controls at very-high frequencies 12.5, 14, and 16 kHz). Tinnitus participants had their tinnitus matched in frequency with a reliable psychoacoustic method (68) and the most reported dominant frequencies were 11 and 16 kHz. Results showed that besides an increased startle response, there was indeed a GPIAS deficit in tinnitus participants, but in both 500 and 4 kHz background noises. Yet, barely half of the participants reported 4 kHz as contributing to their tinnitus, and only one person reported having 500 Hz as contributing to the tinnitus. In contrast, there was no deficit in prepulse inhibition compared to controls without tinnitus. These findings were essentially replicated in a subset of participants 6 months later. These results are not incompatible with animal data but along with other studies (39, 67), cast a serious doubt on the interpretation that tinnitus “fills-in” the gap, since there was no correspondence between the tinnitus and the background noise frequencies.

Mahmoudian et al. (50) examined the cortical correlates of gap detection in tinnitus participants and matched controls using electroencephalography (EEG). More specifically, the mismatched negativity (MMN) waves between standard and deviant sounds were compared between tinnitus and controls without tinnitus. The auditory MMN response is an auditory evoked potential that in response to a deviant stimulus embedded in a train of standard stimuli. It can be elicited by any discriminable change in a sound sequence irrespective of the subject’s attention and therefore probably reflects pre-attentive stages of sound processing. Trains of 75 ms pure tones at 500, 1 and 1.5 kHz (standard stimuli) were presented at 65 dB SPL and 7-ms gaps were inserted occasionally within these pure tones (deviant stimuli). MMN amplitudes for the gap detection stimuli were significantly smaller in the Tinnitus group compared to controls, suggesting a cortical deficit to process short gaps. Interestingly, had authors applied the appropriate Bonferroni corrections for multiple *t*-tests, gap-related MMN amplitude differences would have remained significant while the other deviant types (frequency, intensity, duration, and location) would have not? Hearing thresholds at 500, 1 and 1.5 kHz did not differ between groups. However, there is no information about the relationship between gap deficits and tinnitus or background pure-tone frequencies.

Finally, using the Gap-in-noise test (69) involving detecting short gaps (2–20 ms) within a 50 dB SL white noise background, Mehdizade Gilani et al. (51) reported greater thresholds and lower performance in both ears for normal hearing tinnitus patients compared to controls without tinnitus. No information was provided about the relationship between these findings and tinnitus characteristics.

In contrast, two further studies found no-gap detection deficits in tinnitus. Using a gap duration of 50 ms but a GO/NO-GO gap detection psychophysical task rather than GPIAS, Campolo et al. (49) found no-gap detection deficit. Authors hypothesized that if tinnitus fills-in the gap, tinnitus participants would have difficulty to detect a gap embedded in a noise tuned to their tinnitus frequency, but should be able to detect silent gaps in background noises above or below their (carefully matched) tinnitus frequency. Various 90 s, 15 dB SL narrow-band noises in which 50 ms gaps were inserted were presented and participants had to press a key when detecting a gap and refrain from doing so when there was no gap. Tinnitus participants had no deficit to detect gaps in noises that were 1 octave below, above, or at their tinnitus frequency, and detection in these conditions did not differ from one another. Overall, however, tinnitus participants were impaired in detecting gaps compared to controls, significantly in the left ear (95.5 vs. 86.8%), but not in the right ear (93 vs. 88.6%). This effect was attributed to hearing loss that was more pronounced in tinnitus than in non-tinnitus participants.

Boyen et al. (52) also used a psychophysical gap detection task but smaller gap durations. Four 300 ms narrow-band noise (4–8, 4–5, 5–6.3, 6.3–8 kHz) served as stimuli and were presented at 5, 10, and 25 dB SL for each participant. The task was an adaptive 3I-3AFC where a gap was inserted within one of three noise stimuli, starting with 30 ms gaps and decreasing in duration with a two-down, one-up procedure. Feedback was provided after each trial. Results showed that there were no differences in gap detection thresholds in tinnitus participants compared to no-tinnitus participants matched in age, gender, and hearing loss or to a control group of non-tinnitus young participants without hearing loss. In addition, there was no relation between the matched tinnitus frequency and the gap detection.

In summary, neither gap duration – 50 ms in Ref. (48, 49) <10 ms in Ref. (50–52) – nor hearing thresholds – comparable between groups in Ref. (48, 50, 52), not comparable between groups in Ref. (49); unknown in Ref. (51) – seem to explain differences in findings among human studies. However, two other factors might be involved, namely, task requirements and background noise levels. Indeed, two studies (49, 52) that used tasks requiring high-level cognitive resources found not gap detection deficits [an exception is the study of Mehdizade Gilani et al. (51)] whereas the two studies (48, 50) that used tasks not requiring such resources, namely startle reflex and pre-attentional processing, found gap detection deficits although not specific to the tinnitus frequency. It is possible that the requirement – or lack thereof – of attentional resources on a simple task, that is, behavioral performance in detecting a gap, can overcome subtle gap deficits. Perhaps more importantly, studies that found gap deficits used background noise levels higher (50 dB SL and 65 dB SPL) than those that did not (between 5 and 25 dB SL). The type of neurons responding to low vs. high-level sounds might be involved.

## Future Research Directions

The GPIAS method is based on the assumption that tinnitus “fills-in” the gap during GPIAS testing (12). However, several recent studies conducted on laboratory animals as well as on humans found little evidence to support this assumption. While these studies raised concerns and emphasized caution, they did not rule out a possibility that GPIAS deficits can indeed be interpreted as an indication of tinnitus. On the other hand, multiple studies have demonstrated robust GPIAS deficits in a subset of animals after manipulations that are known to induce tinnitus. Such deficits have been shown to persist for a long time period (a 7-month to 1-year) after being first identified (36, 37). The presence of tinnitus identified by the GPIAS method has been supported by two studies (26, 36). Furthermore, changes in neuronal firing rate and synchrony in the range of tinnitus assessed by GPIAS or altered latencies and amplitudes of the selected ABR waveforms, have shown to be linked to GPIAS deficits (42, 43). Further GPIAS research is needed to clarify whether these deficits are linked to tinnitus or some other auditory abnormalities. Human studies would be the most logical and convincing way to test whether the GPIAS method can reliably and objectively assess tinnitus simply because we have control of the tinnitus percept in humans. One line of research would be to use EEG to collect evoked potentials while manipulating acoustic parameters surrounding the gap. Background noise levels (high vs. low), frequency (tinnitus frequency vs. frequency above or below), and gap duration (short vs. long) should be manipulated in order to disentangle which of these parameters is the most critical to explain discrepancies among studies. Another line would be to use the startle paradigm and manipulate the background frequency and sound levels for a single individual to discover whether particular gap deficits are correlated with the tinnitus frequency. In addition, since tinnitus is a type of subjective experience, it is of importance to develop new experimental paradigms with the manipulation of attention to either the (internal) tinnitus object or the (external) gap prepulse in order to investigate the potential link between GPIAS deficits and tinnitus [see Ref. (70) for a review].

Although human studies would be the first choice, studies on animals could also make a significant contribution to answer this fundamental question. It would be useful to confirm the presence of tinnitus identified by GPIAS with other behavioral paradigms that are known to involve the auditory cortex, rather than the GPIAS paradigm that relies mostly on the acoustic startle brain stem circuit. The contribution of hearing loss to the GPIAS deficits also needs to be further clarified and, if possible, ruled out. Generalized hearing loss can attenuate startle responses while frequency specific hearing deficits could render gaps preceding a startle in an otherwise continuous background less audible (8, 38).

The tinnitus “filling-in” assumption has been challenged in several animal and human studies. However, if animals or humans constantly experience a phantom sound, this sound must still be present during the silent gap during GPIAS testing. However, a gap, even partially filled by tinnitus, would still be detectible unless the background sound used in the testing was ideally matched to the intensity and spectrum of the tinnitus. Such matching process could be possible for humans but would be a dubious process for animal due to a lack of knowledge about their tinnitus characteristics. Contrary to what has been commonly assumed in animal research tinnitus is rarely composed of only one frequency but rather encompasses a wide range of frequencies. Given the right methodology (i.e., finding tinnitus spectrum and level), such hypotheses can be relatively easy to explore with tinnitus patients but very challenging with animal models. One of the possible approaches would be to create a psychometric function representing the ability of a tinnitus patient to identify a gap based on various background noise and gap parameters. Ideally, one would identify a range of this function that allows for optimal tinnitus detection. A similar approach has recently been suggested to select appropriate startle stimulus intensity in mice (58). Startle input–output function has sigmoid shape. At high startle intensities (i.e., 120 dB SPL), this function is saturated. As a result, changes in gap detection deficits are hardly detectible if the startle stimulus is 120 dB SPL. However, when the startle stimulus intensity was reduced by about 25% of the maximum, such startle response was very sensitive to small changes in gap detection [see Figure 8 in Ref. (58)]. If all stimulus parameters can be adjusted in the similar way, it might greatly improve the sensitivity of the GPIAS method. In the long-term, the development of new techniques such as optogenetic, which uses light to control and read out specific neurons, might answer questions related to what types of neurons (61) are involved in gap detection deficits in tinnitus.

## Conflict of Interest Statement

The authors declare that the research was conducted in the absence of any commercial or financial relationships that could be construed as a potential conflict of interest.
